# Factors associated with mortality in HIV-infected people in rural and urban South Africa

**DOI:** 10.3402/gha.v7.25488

**Published:** 2014-09-29

**Authors:** Kennedy N. Otwombe, Max Petzold, Tebogo Modisenyane, Neil A. Martinson, Tobias Chirwa

**Affiliations:** 1 Perinatal HIV Research Unit, Faculty of Health Sciences, University of the Witwatersrand, Johannesburg, South Africa; 2 School of Public Health, Faculty of Health Sciences, University of the Witwatersrand, Johannesburg, South Africa; 3 Centre for Applied Biostatistics, Occupational and Environmental Medicine, University of Gothenburg, Gothenburg, Sweden; 4 Center for TB Research, Johns Hopkins University, Baltimore, MD, USA

**Keywords:** HIV, Mortality, Rural, Urban, Frailty, HAART

## Abstract

**Background:**

Factors associated with mortality in HIV-infected people in sub-Saharan Africa are widely reported. However rural–urban disparities and their association with all-cause mortality remain unclear. Furthermore, commonly used classical Cox regression ignores unmeasured variables and frailty.

**Objective:**

To incorporate frailty in assessing factors associated with mortality in HIV-infected people in rural and urban South Africa.

**Design:**

Using data from a prospective cohort following 6,690 HIV-infected participants from Soweto (urban) and Mpumalanga (rural) enrolled from 2003 to 2010; covariates of mortality were assessed by the integrated nested Laplace approximation method.

**Results:**

We enrolled 2,221 (33%) rural and 4,469 (67%) urban participants of whom 1,555 (70%) and 3,480 (78%) were females respectively. Median age (IQR) was 36.4 (31.0–44.1) in rural and 32.7 (28.2–38.1) in the urban participants. The mortality rate per 100 person-years was 11 (9.7–12.5) and 4 (3.6–4.5) in the rural and urban participants, respectively. Compared to those not on HAART, rural participants had a reduced risk of mortality if on HAART for 6–12 (HR: 0.20, 95% CI: 0.10–0.39) and >12 months (HR: 0.10, 95% CI: 0.05–0.18). Relative to those not on HAART, urban participants had a lower risk if on HAART >12 months (HR: 0.35, 95% CI: 0.27–0.46).

The frailty variance was significant and >1 in rural participants indicating more heterogeneity. Similarly it was significant but <1 in the urban participants indicating less heterogeneity.

**Conclusion:**

The frailty model findings suggest an elevated risk of mortality in rural participants relative to the urban participants potentially due to unmeasured variables that could be biological, socio–economic, or healthcare related. Use of robust methods that optimise data and account for unmeasured variables could be helpful in assessing the effect of unknown risk factors thus improving patient management and care in South Africa and elsewhere.

South Africa has the highest number of people living with HIV and the largest antiretroviral treatment (ART) programme globally (1). Driven by the need to achieve the millennium development goal of increasing the number of HIV-infected people accessing treatment (2), over 2 million people have been started on ART with another half million planned to be started each year. Mortality in HIV-infected individuals is high (3, 4), especially prior to ART treatment and while awaiting ART (5). This is associated with highly immunosuppressed patients with low CD4 counts and high viral loads. While survival on ART is markedly improved, mortality rates in the early stages of treatment remain relatively high (4, 6) signifying the importance of immunosuppression at the time of ART initiation. Knowledge of risk factors for mortality is important to improve ART programmes and to prolong life. Factors associated with mortality in HIV-infected people in sub-Saharan Africa are widely reported with variations. They include CD4 and total lymphocyte counts, HIV RNA level, WHO and CDC staging, anaemia, malnutrition, and gender amongst others (6–11). However, prior studies do not specifically report and compare mortality in rural and urban settings. Furthermore time-to-event methods remain the most commonly used for the investigation of mortality risk factors particularly in HIV-infected cohorts (12). Cox proportional hazards regression analysis, the most popular approach assumes that the risk of mortality is homogeneous (13), an assumption that does not often hold (14). In epidemiological studies where some participants are related either genetically or are from the same family, share some unmeasured covariates, or have multiple recurrence events, their survival times are not independent due to within -subject dependency (15). Analysis of such data requires use of a method that accounts for dependence leading to heterogeneity such as frailty modelling (14–16), an extension of the Cox regression. Frailty modelling incorporates within-subject dependency by considering that the individual risk of events differs between subjects through accounting for unmeasured variables (15–17). Adjusting for within-subject correlation and accounting for unmeasured variables are some of the advantages of frailty modelling. Disadvantages include handling of competing risks and assuming the same risk within a cluster of unobserved variables.

Understanding the dynamics of mortality in HIV-infected people in sub-Saharan Africa is important as it influences patient management and care. As such, assessment of mortality surveillance patterns requires the use of robust statistical analysis methods that will optimise findings leading to appropriate policy decisions from evidence-based research. While current methods are well established, recent approaches, though more complex, provide further insight through their ability to model dependency. Detailed research from sub-Saharan Africa comparing factors associated with mortality in HIV-infected people between rural and urban areas using recently developed advanced methods are limited. Furthermore, those employing methods that account for unmeasured variables in this setting are not properly described. And, more importantly, we hypothesise that they inadequately capture uncertainty and are less precise. We therefore sought to assess and compare factors associated with mortality in HIV-infected adults in a large cohort of participants in a wellness programme in rural and urban South Africa while accounting for unmeasured variables in both sites.

## Methods

### Setting and study population

Data from two South African study sites were used: Perinatal HIV Research Unit (PHRU) in Soweto and Tintswalo hospital in Mpumalanga. In this study, participants enrolled in PHRU were classified as urban while those enrolled in Tintswalo were classified as rural. While both sites provided treatment using the same guideline from the department of health and similar resources such as nurses with similar qualifications, Tintswalo hospital is a primary health centre while the PHRU clinic is a research centre.

Soweto is an urban area in the city of Johannesburg in Gauteng, South Africa, with a population greater than a million people (18). It is in the southwestern part of Johannesburg where approximately 40% of the population resides. While many households are poor with high unemployment rates, there is a mix of poor and wealthy residents (18).

Tintswalo hospital is situated in the Bushbuckridge district of rural South Africa’s northeast with a population of 541,247 people. Bushbuckridge is among the poorest areas of South Africa with up to 75% of the population living in poverty (19).

A nurse-based wellness programme was established in PHRU and Tinstwalo to provide pre- and post-treatment care for HIV-infected adults between the years 2003 and 2010. Participants in the study were referred from voluntary counselling and testing centres, surrounding clinics, hospital wards, or research programmes. Referrals for ART were made for those eligible, based on the South African ART treatment guidelines at the time (CD4 cell count <200 cells/mm^3^) (20). Inclusion criteria were diagnosis of HIV, 18 years of age or older, receiving primary clinical care at PHRU or Tintswalo clinics and providing written informed consent. Further study procedures are presented elsewhere (21, 22).

Study questionnaires were administered at baseline and follow-up visits that occurred every 6 months. Losses to follow-up were minimised through actively following up participants by way of telephone calls, letters, and then home visits. Weight, CD4 counts, creatinine, and haemoglobin estimations were measured at scheduled six monthly visits while other investigations were done according to clinical presentation. To be considered positive for tuberculosis (TB), subjects had to meet at least one of the following criteria (21): initiation of multidrug TB therapy, presence of acid fast bacilli on microscopy or a biopsy suggestive of TB, mycobacterium culture positive for acid fast bacilli or *Mycobacterium tuberculosis*, hospitalisation due to TB or cause of death ascribed to TB.

### Measures

For this study, the outcome measure was all-cause mortality, primarily through making contact with the next of kin. Socio-demographic measures included age, household income, number of people and rooms in a household, ever smoked and ever employed. Others were height and weight for calculating body mass index (BMI), HAART status, ever had TB and CD4 counts. BMI was categorised into three groups using the CDC classification: underweight (<18.5), normal (18.5–24.9), or overweight/obese (≥25) while CD4 count was categorised into 0–200, 201–350, 351–500, and >500 cells/mm^3^.

### Ethics

Ethical approval was provided by the Human Research Ethics Committee of the University of the Witwatersrand.

### Statistical analysis

Data were stratified by site. Continuous measures at enrolment such as age, BMI, and CD4 count were assessed descriptively by median and interquartile ranges and compared between the two groups using the Kruskal–Wallis non-parametric test. Frequencies and associated proportions were determined for categorical measures and compared using the Fishers Exact and Chi--square analysis as appropriate.

Mortality rates (95% confidence interval) per 100 person-years were determined using Poisson regression modelling by site for different lengths of time pre- and post-HAART and presented graphically. The Kaplan–Meier test was used to determine differences in surveillance patterns by site.

Factors associated with mortality by site were assessed using the integrated nested Laplace approximation (INLA) (23, 24), where univariate and multivariate shared gamma frailty models were fitted. INLA is a Bayesian inference technique, computationally efficient due to its speed relative to existing methods and more accurate parameter estimates. For the purpose of interpretation, posterior Bayesian estimates generated from INLA were exponentiated to determine their approximate hazard ratios and 95% confidence intervals (25).

INLA accounted for unmeasured variables and allowed for the comparison of frailty variances between sites. A significant frailty variance with a value >1 suggests a higher rate for the event (or shorter survival times) than would be predicted under the basic Cox model while <1 suggests a lower rate (longer survival times). Hence, failure to account for frailty may overestimate or underestimate the hazard rate.

Backward selection together with inclusion of plausible socio-demographic and clinical factors was used to determine the final multivariate model. Socio-demographic variables included gender, ever employed, and ever smoked. Clinical variables included HAART status, BMI, CD4 cell count, and ever had TB. The most recent information was used for HAART status while BMI and CD4 cell count values were considered over time.

A 95% confidence interval excluding one was determined as significant. Model fit was assessed using the deviance information criterion.

Multiple imputation (MI) under the missing-at-random mechanism was used to impute 18% (n=1,195) and 11% (n=704) of missing CD4 count and BMI data, respectively (26–28). Five imputed datasets reflecting different scenarios were generated and analysed following the MI procedure.

Several sensitivity analyses were performed using INLA. This included assessing factors associated with mortality overall, pre- and post-HAART. Kaplan–Meier plots of time to death by gender, HAART use, CD4 count, WHO staging, and BMI were also fitted. For participants that were lost to follow-up, their time in the study up to their last visit was included in the analysis.

All statistical analyses were performed using R and SAS Enterprise Guide 5.1 under the assumption of a two-sided test at 5% significance level.

## Results

Overall, 6,690 participants were enrolled and followed up for a cumulative total of 2225.8 and 8185.9 person-years in the rural (33%) and urban (67%) sites, respectively. The median (IQR) pre- and post-HAART time in years was 0.6 (0–1.9) and 1.1 (0.5–2.6), respectively. Overall, 75% were women: rural (70%) and urban (78%). The median age at enrolment was 36.4 (IQR: 31.0–44.1) and 32.7 (IQR: 28.2–38.1) years for rural and urban sites, respectively.

Rural participants were more likely to report a household income of ≤R1,000 per month compared to those in the urban areas (72.8% vs. 48.5%; *p*<0.0001). The proportion not on HAART at the end of the study was significantly higher in the urban area compared to the rural area (63.2% vs. 30.0%; *p*<0.0001) while unemployment was significantly higher in the rural area (86.7% vs. 70.7%; *p*<0.0001) relative to the urban areas.

At enrolment, 54% of the rural and 27% of the urban participants had either started HAART or were already on HAART. On follow-up, 17 and 10% of rural and urban participants, respectively, initiated HAART.

The median BMI of urban participants was higher than that of the rural participants (24.9 vs. 21.0; *p*<0.0001). Similarly, the median CD4 count at entry of rural participants was lower than that in the urban participants (178 cells/mm^3^ vs. 303 cells/mm^3^; *p*<0.0001, Table 1). The proportion on HAART for <6 months and with a most recent CD4 count <200 cells/mm^3^ was significantly higher in the urban compared to the rural participants (76% vs. 59%; *p*<0.0001). Similarly, urban participants on HAART for 6–12 months and with a most recent CD4 count <200 cells/mm^3^ were significantly more than in the rural participants (69% vs. 53%; *p*=0.0004). The proportion unemployed and with a CD4 count <200 cells/mm^3^ was significantly higher in the rural participants compared to the urban participants (40% vs. 24%; *p*<0.0001). The proportion ever with TB in the rural participants was significantly higher than in the urban participants (44.8% vs. 25.4%; *p*<0.0001, Table 2).

**Table 1:** Participant characteristics at enrolment by site

	Rural			Urban		
	
Variable	Overall (n=2,221)	Male (n=666, 30%)	Female (n=1,555, 70%)	Overall (n=4,469)	Male (n=989, 22%)	Female (n=3,480, 78%)
Median age (IQR) in years	36.4 (31.0–44.1)	39.2 (33.3–48.1)	35.2 (30.0–42.5)	32.7 (28.2–38.1)	35.7 (31.5–41.5)	31.7 (27.4–37.0)
Age group in years						
18–25 (%)	216 (9.7)	30 (4.5)	186 (12.0)	672 (15.0)	65 (6.6)	607 (17.4)
26–30 (%)	342 (15.4)	72 (10.8)	270 (17.4)	1,117 (25.0)	158 (16.0)	959 (27.6)
31–34 (%)	420 (18.9)	111 (16.7)	309 (19.9)	984 (22.0)	222 (22.4)	762 (21.9)
35–39 (%)	424 (19.1)	136 (20.4)	288 (18.5)	864 (19.3)	250 (25.3)	614 (17.6)
>40 (%)	819 (36.9)	317 (47.6)	502 (32.3)	832 (18.6)	294 (29.7)	538 (15.5)
Median CD4 count (IQR)	178.0 (81.0–333.6)	162.0 (69.0–289.9)	184.0 (86.0–350.6)	303.0 (156.0–470.7)	281.0 (142.0–443.7)	309.0 (163.0–478.2)
CD4 categories						
0–200 (%)	1,235 (55.6)	398 (59.8)	837 (53.8)	1,474 (33.0)	367 (37.1)	1,107 (31.8)
201–350 (%)	472 (21.3)	144 (21.6)	328 (21.1)	1,102 (24.7)	241 (24.4)	861 (24.7)
351–500 (%)	284 (12.8)	75 (11.3)	209 (13.4)	915 (20.5)	190 (19.2)	725 (20.8)
>500 (%)	230 (10.4)	49 (7.4)	181 (11.6)	978 (21.9)	191 (19.3)	787 (22.6)
Median BMI (IQR)	21.0 (17.9–25.0)	19.9 (17.2–23.4)	21.4 (18.4–25.8)	24.9 (20.7–29.2)	22.5 (18.2–26.4)	25.6 (21.4–30.0)
BMI						
Underweight (%)	638 (29.0)	241 (36.9)	397 (25.7)	594 (13.4)	240 (24.8)	354 (10.2)
Normal (%)	987 (44.9)	299 (45.7)	688 (44.6)	1,612 (36.4)	393 (40.6)	1,219 (35.2)
Obese/overweight (%)	572 (26.0)	114 (17.4)	458 (29.7)	2,226 (50.2)	336 (34.7)	1,890 (54.6)
Median crowding index (IQR)	1.5 (1.0–2.3)	1.3 (0.8–2.0)	1.6 (1.0–2.5)	1.5 (1.0–2.3)	1.0 (1.0–2.0)	1.5 (1.0–2.5)
Median household income (IQR) in Rands	600.0 (189.8–1010.0)	780.0 (50.0–1500.0)	500.0 (190.0–974.7)	1080.0 (500.0–2130.0)	1200.0 (600.0–2400.0)	1020.0 (500.0–2020.0)
Household income						
≤R1,000 (%)	1,516 (72.8)	423 (67.4)	1,093 (75.2)	2,161 (48.5)	453 (45.9)	1,708 (49.2)
R1,001–R5,000 (%)	528 (25.4)	193 (30.7)	335 (23.1)	2,092 (46.9)	487 (49.3)	1,605 (46.3)
>R5,000 (%)	37 (1.8)	12 (1.9)	25 (1.7)	203 (4.6)	47 (4.8)	156 (4.5)

BMI, body mass index; HAART, highly active antiretroviral therapy; TB, tuberculosis.

Crowding index is the number of people divided by the number of rooms in a household; household income refers to household monthly income; for household income, 1USD was equivalent to R7 at the time of the study.

**Table 2:** Distribution of HAART status, employment, smoking, and TB by site

	Rural			Urban		
	
Variable	Overall (n=2,221)	Male (n=666, 30%)	Female (n=1,555, 70%)	Overall (n=4,469)	Male (n=989, 22%)	Female (n=3,480, 78%)
Time on HAART						
No HAART (%)	666 (30.0)	174 (26.1)	492 (31.6)	2,826 (63.2)	555 (56.1)	2,271 (65.3)
<6 months (%)	468 (21.0)	153 (23.0)	315 (20.3)	356 (8.0)	108 (10.9)	248 (7.1)
6–12 months (%)	434 (20.0)	133 (20.0)	301 (19.4)	178 (4.0)	35 (3.5)	143 (4.1)
>12 months (%)	653 (29.0)	206 (30.9)	447 (28.7)	1,109 (24.8)	291 (29.5)	818 (23.5)
Ever employed						
Yes (%)	296 (13.3)	143 (21.5)	153 (9.8)	1,309 (29.3)	400 (40.4)	909 (26.1)
No (%)	1,925 (86.7)	523 (78.5)	1,402 (90.2)	3,160 (70.7)	589 (59.6)	2,571 (73.9)
Ever smoked						
Yes (%)	358 (16.1)	295 (44.3)	63 (4.1)	1,284 (28.7)	706 (71.4)	578 (16.6)
No (%)	1,863 (83.9)	371 (55.7)	1,492 (95.9)	3,185 (71.3)	283 (28.6)	2,902 (83.4)
Ever had TB						
Yes (%)	995 (44.8)	350 (52.6)	645 (41.5)	1,134 (25.4)	313 (31.6)	821 (23.6)
No (%)	1,226 (55.2)	316 (47.4)	910 (58.5)	3,335 (74.6)	676 (68.4)	2,659 (76.4)

The most recent value is provided for time on HAART.

**Fig. 1:**
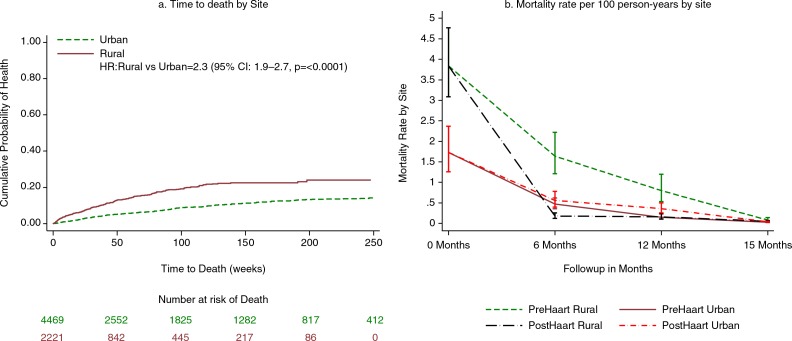
Survival and mortality rate plots by site. a) displays the cumulative probability of death by site in the cohort of HIV-infected people in South Africa. b) is a plot of mortality rates during follow-up by site showing the period prior to initiating HAART to post-HAART.

Overall, 566 (8%) of the participants died during follow-up: 11 and 7% in rural and urban sites, respectively (*p*<0.0001). Of these, majority were females (63% rural vs. 69% urban; p=0.1952). Overall, the mortality rate per 100 person-years in rural sites was higher at 11 (95% CI: 9.7–12.5) compared to urban sites at 4 (95% CI: 3.6–4.5). In the first 6 months, 48% of rural participants had died while 51% of the deaths in the urban site occurred after 12 months. Mortality rates per 100 person-years while on HAART were 11 (95% CI: 8.7–14.0) and 7 (95% CI: 5.9–8.3) for rural and urban participants, respectively. When taking HAART, majority of the deaths in the rural site occurred within the first 3 months of treatment initiation (49%) whereas they occurred after 12 months in the urban site (44%). Further description of those who died pre- and post-treatment in the two sites by CD4 category and BMI is presented in Table 3. Relative to the urban participants, mortality rates in the rural participants was higher (Fig. 1).

**Table 3:** Characteristics of those who died

	Rural			Urban		
	
Variable	Overall	Male	Female	Overall	Male	Female
Total deaths	242	88	154	324	101	223
Mortality rate per 100 person-years (95% CI)	11 (9.7–12.5)	14 (11.4–17.3)	10 (8.5–11.7)	4 (3.6–4.5)	6 (4.9–7.3)	3 (2.6–3.4)
Time to death in months						
0–3 (%)	76 (31.5)	30 (34.1)	46 (30.1)	38 (12.1)	17 (17.7)	21 (9.6)
3–6 (%)	40 (16.6)	16 (18.2)	24 (15.7)	47 (15.0)	16 (16.7)	31 (14.2)
6–9 (%)	32 (13.3)	10 (11.4)	22 (14.4)	36 (11.5)	9 (9.4)	27 (12.4)
9–12 (%)	26 (10.8)	10 (11.4)	16 (10.5)	27 (8.6)	5 (5.2)	22 (10.1)
>12 (%)	67 (27.8)	22 (25)	45 (29.4)	166 (52.9)	49 (51)	117 (53.7)
CD4 categories						
0–200 (%)	167 (69.0)	59 (67.0)	108 (70.1)	195 (60.2)	66 (65.3)	129 (57.8)
201–350 (%)	35 (14.5)	11 (12.5)	24 (15.6)	69 (21.3)	18 (17.8)	51 (22.9)
351–500 (%)	24 (9.9)	11 (12.5)	13 (8.4)	36 (11.1)	11 (10.9)	25 (11.2)
>500 (%)	16 (6.6)	7 (8.0)	9 (5.8)	24 (7.4)	6 (5.9)	18 (8.1)
BMI						
Underweight (%)	109 (45.2)	41 (46.6)	68 (44.7)	75 (23.6)	34 (35.1)	41 (18.6)
Normal (%)	95 (39.6)	37 (42.0)	58 (38.2)	126 (39.6)	40 (41.2)	86 (38.9)
Overweight/obese (%)	36 (15.0)	10 (11.4)	26 (17.1)	117 (36.8)	23 (23.7)	94 (42.5)
Deaths on HAART	68	31	37	133	52	81
Mortality rate per 100 person-years (95% CI)	11 (8.7–14.0)	15 (10.5–21.3)	9 (6.5–12.4)	7 (5.9–8.3)	9 (6.9–11.8)	6 (4.8–7.5)
Time to death in months						
0–3 (%)	33 (48.5)	15 (48.4)	18 (48.6)	21 (16.4)	10 (20.4)	11 (13.9)
3–6 (%)	12 (7.6)	5 (16.1)	7 (18.9)	17 (13.3)	8 (16.3)	9 (11.4)
6–9 (%)	9 (13.2)	3 (9.7)	6 (16.2)	18 (14.1)	6 (12.2)	12 (15.2)
9–12 (%)	4 (5.9)	2 (6.5)	2 (5.4)	13 (10.2)	2 (4.1)	11 (13.9)
>12 (%)	10 (14.7)	6 (19.4)	4 (10.8)	59 (46.1)	23 (46.9)	36 (45.6)
CD4 categories						
0–200 (%)	43 (63.2)	19 (16.3)	24 (64.9)	86 (64.7)	39 (75.0)	47 (58.0)
201–350 (%)	11 (6.2)	3 (9.7)	8 (21.6)	23 (17.3)	6 (11.5)	17 (21.0)
351–500 (%)	9 (13.2)	6 (19.4)	3 (8.1)	13 (9.8)	3 (5.8)	10 (12.3)
>500 (%)	5 (7.4)	3 (9.7)	2 (5.4)	11 (8.3)	4 (7.7)	7 (8.6)
BMI						
Underweight (%)	25 (37.3)	13 (41.9)	12 (33.3)	30 (22.9)	19 (38.0)	11 (13.6)
Normal (%)	24 (35.8)	9 (29.0)	15 (41.7)	48 (36.6)	21 (42.0)	27 (33.3)
Overweight/Obese (%)	18 (26.9)	9 (29.0)	9 (25.0)	53 (40.5)	10 (20.0)	43 (53.1)

In the rural participants, being on HAART for 6–12 months (HR: 0.20, 95% CI: 0.10–0.39) and >12 months (HR: 0.10, 95% CI: 0.05–0.18) relative to no HAART was protective after adjusting for CD4 count (Table 4). However, underweight BMI compared to normal BMI was associated with a higher risk of mortality (HR: 1.74, 95% CI: 1.06–2.85).

**Table 4:** Factors associated with mortality in rural and urban South Africa

	Rural		Urban	
	
	Univariate	Multivariate	Univariate	Multivariate
	
Variable	HR (95% CI)	HR (95% CI)	HR (95% CI)	HR (95% CI)
Gender				
Female vs. male	0.69 (0.45–1.1)	0.78 (0.48–1.27)	0.53 (0.42–0.67)	1.09 (0.80–1.44)
Time on HAART				
<6 months vs. No HAART	1.12 (0.56–2.22)	1.16 (0.62–2.16)	17.25 (11.74–24.78)	**4.48 (3.0**–**6.56)**
6–12 months vs. No HAART	0.17 (0.08–0.37)	**0.20 (0.10**–**0.39)**	7.20 (4.89–10.35)	**2.39 (1.60**–**3.47)**
>12 months vs. No HAART	0.10 (0.05–0.20)	**0.10 (0.05**–**0.18)**	1.03 (0.80–1.32)	**0.35 (0.27**–**0.46)**
BMI				
Underweight vs. normal	2.20 (1.40–3.24)	**1.74 (1.06**–**2.85)**	4.29 (3.26–5.62)	**2.49 (1.87**–**3.11)**
Overweight/obese vs. normal	0.55 (0.30–0.98)	0.61 (0.31–1.16)	0.55 (0.42–0.71)	**0.73 (0.56**–**0.90)**
CD4 Count (cells/mm^3^)				
201–350 vs. 0–200	0.26 (0.11–0.56)	**0.25 (0.13**–**0.47)**	0.19 (0.14–0.24)	**0.22 (0.16**–**0.29)**
351–500 vs. 0–200	0.49 (0.20–1.08)	**0.44 (0.21**–**0.89)**	0.09 (0.06–0.13)	**0.11 (0.07**–**0.17)**
>500 vs. 0–200	0.25 (0.08–0.66)	**0.22 (0.09**–**0.53)**	0.04 (0.02–0.08)	**0.05 (0.03**–**0.09)**
Ever employed				
No vs. Yes	0.95 (0.53–1.83)	1.13 (0.58–2.24)	2.03 (1.55–2.70)	**1.87 (1.42**–**2.48)**
Ever smoked				
Yes vs. No	0.99 (0.56–1.56)		2.07 (1.66–2.59)	**1.37 (1.04**–**1.79)**
Ever had TB				
Yes vs. No	1.66 (1.10–2.47)	1.55 (0.98–2.46)	2.84 (2.29–3.54)	**2.76 (2.19**–**3.48)**

Bold entries represent significant values.

Being on HAART for <6 months (HR: 4.48, 95% C: 3.0–6.56) and 6–12 months (HR: 2.39, 95% CI: 1.60–3.47) compared to no HAART was associated with an elevated risk of mortality in the urban participants after adjusting for CD4 count, employment, smoking, and TB history. However, those with overweight/obese BMI (HR: 0.73, 95% CI: 0.56–0.90) had a significantly lower risk of mortality compared to those with normal BMI.

The estimated frailty variances were statistically significant for rural and urban sites; 5.65 (95% CI: 4.50–7.19) and 5.4×10^−5^ (95% CI: 1.5×10^−5^ – 8.0×10^−4^) respectively.

## Discussion

In sub-Saharan Africa where the burden of HIV is the greatest, research on factors associated with all-cause mortality in HIV-infected cohorts has widely been done. However, research on risk factors using advanced Bayesian methods such as INLA that incorporate unmeasured variables is limited (23, 24). Common protective factors against mortality included HAART use for >12 months and CD4 count above 200 cells/mm^3^ while underweight BMI was positively associated with mortality. Even with differences between the rural and urban participants, the hazard rates were often in the same direction.

While both frailty variances were significant, our findings show that the frailty variance for participants in the rural site was higher than that of the urban. This suggests important unmeasured variables relevant to participants in the rural site may not have been considered (as the variance was >1) signifying more heterogeneity (variability). In contrast, the variance for participants in the urban site was <1 signifying less heterogeneity and probably that the model accounted for most of the important variables. Available literature on covariates of mortality relies on classical statistical methods that do not account for unmeasured variables (12). Potential unmeasured variables include 1) biological factors such as undiagnosed TB and/or other opportunistic infections such as cryptococcosis, 2) socio-economic factors that were not included in the data collection process, 3) healthcare-related factors, 4) adherence to HAART medication, and 5) cultural and religious beliefs. A study from Portugal assessed predictors of mortality in HIV-associated hospitalisations, and through frailty modelling, it was shown how unmeasured variables in the form of quality in healthcare in different hospitals affected mortality (17). Hence, an assessment that allows for the use of a mixed methods approach may provide an opportunity for further insight of the unmeasured variables that may qualify as risk factors.

Underweight BMI was associated with mortality in both rural and urban areas with a slightly higher hazard in the urban group. Our findings concur with others from previous studies (5, 21, 29). While overweight or obese BMI was not associated with mortality in rural participants, it was associated with decreased mortality in the urban participants. Previous studies have shown that overweight or obese BMI reduces the risk of mortality (21). The proportion of participants with underweight BMI was higher in the rural group while that of overweight or obese participants was higher in the urban group suggesting differences in nutrition between the two groups. It may also be that the use of HAART mediated in the lack of association between mortality and overweight or obese BMI in the rural participants.

Higher, most recent CD4 count was protective in both groups as similarly reported in previous studies (30, 31). However at enrolment, rural participants had a lower median CD4 count that qualified them to initiate therapy as per the treatment guidelines at the time. As in many treatment programmes across sub-Saharan Africa, first time testers often present for care with low CD4 count. Previous studies have underscored the relationship between treatment initiation, low CD4 count, and early mortality (32–35).

Those on treatment for >6 months had a lower risk of mortality in the rural participants compared to >12 months in the urban participants. Among those on treatment for 6–12 months in the urban participants, a greater proportion had a most recent CD4 count <200 cells/mm^3^. Findings from prior research show an elevated risk of early mortality especially in immunocompromised patients in the early period of treatment (4, 9, 34, 36, 37). The high mortality rate in urban participants may be due to late initiation of treatment leading to advanced HIV disease. Furthermore, the association between late initiation of treatment and low CD4 count is well established. It may also be that mortality was accelerated in this group by opportunistic infections that were not identified in time including undiagnosed and untreated TB. TB remains the most common opportunistic infection diagnosed in HIV-infected people.

Unemployed urban participants had a higher risk of mortality, a finding that concurs with previous research (38–41). HIV care was provided at no cost in both sites irrespective of their employment status. It maybe that mortality is affected by differential access to care in urban participants. Other studies have shown that mortality could be affected by poor social situations such as unemployment, poor housing, and social isolation. Furthermore, these factors may influence one’s health negatively (38, 39).

There was an association between ever suffering from TB and mortality in participants from the urban site. Contrary to our findings, a previous study from Durban in South Africa found no association (36). TB is a common opportunistic infection in HIV-infected individuals and its association with mortality is indisputable (21, 37, 42). Since urban participants were significantly more likely to have CD4 counts <200 cells/mm^3^ up to 12 months on HAART, it may be that they are immune-compromised and more susceptible to opportunistic infections, such as TB, and early mortality.

Some variables such as employment were self-reported and may have been influenced by social desirability bias. Using cumulative variables such as ‘ever smoking’ or ‘ever employed’ may lead to misclassification given the fact that it is the cumulative, rather than the instantaneous. While all attempts were made to collect and report all measures, there were some missing values that were estimated by MIs which may have introduced some bias. Some deaths may have been misclassified since some participants were lost to follow-up and reasons for loss to follow-up were not systematically recorded. However, an advantage of our study was the large sample size and the use of a Bayesian frailty survival model that accounts for unmeasured factors.

## Conclusion

Our findings suggest that using robust advanced statistical analysis methods may provide further insight to risk factors for mortality. Furthermore, and where appropriate, a mixture of methods involving both quantitative and qualitative approaches could be employed allowing for further understanding of the unmeasured variables. Such an approach is likely to inform HIV care managers and policy makers of important factors for which interventions could be developed or existing ones enhanced to improve patient management and care. Any such interventions should alleviate rural–urban healthcare disparities not only in South Africa but also in other countries with similar experiences. Interventions should specifically help in alleviating the challenges faced by rural communities in accessing care.
